# Why are the poor less covered in Ghana’s national health insurance? A critical analysis of policy and practice

**DOI:** 10.1186/s12939-016-0320-1

**Published:** 2016-02-25

**Authors:** Agnes Millicent Kotoh, Sjaak Van der Geest

**Affiliations:** Department of Population, Family and Reproductive Health, School of Public Health, University of Ghana, Legon, Ghana; Department of Sociology and Anthropology, University of Amsterdam, Amsterdam, The Netherlands

**Keywords:** Health insurance, Enrolment, Retention, Poverty, Exemption, Equity, Assurance maladie, Inscription, Rétention, Pauvreté, Exemption, Equité

## Abstract

**Background:**

The National Health Insurance Scheme (NHIS) was introduced in Ghana to ensure equity in healthcare access. Presently, some low and middle income countries including Ghana are using social health insurance schemes to reduce inequity in access to healthcare. In Ghana, the NHIS was introduced to address the problem of inequity in healthcare access in a period that was characterised by user-fee regimes. The premium is heavily subsidised and exemption provided for the poorest, yet studies reveal that they are least enrolled in the scheme. We used a multi-level perspective as conceptual and methodological tool to examine why the NHIS is not reaching the poor as envisaged.

**Methods:**

Fifteen communities in the Central and Eastern Regions of Ghana were surveyed after implementing a 20 months intervention programme aimed at ensuring that community members have adequate knowledge of the NHIS’ principles and benefits and improve enrolment and retention rates. Observation and in-depth interviews were used to gather information about the effects of the intervention in seven selected communities, health facilities and District Health Insurance Schemes in the Central Region.

**Results:**

The results showed a distinct rise in the NHIS’ enrolment among the general population but the poor were less covered. Of the 6790 individuals covered in the survey, less than half (40.3 %) of the population were currently insured in the NHIS and 22.4 % were previously insured. The poorest had the lowest enrolment rate: poorest 17.6 %, poor 31.3 %, rich 46.4 % and richest 44.4 % (p = 0.000). Previous enrolment rates were: poorest (15.4 %) and richest (23.8 %), (p = 0.000). Ironically, the poor’s low enrolment was widely attributed to their poverty. The underlying structural cause, however, was policy makers’ and implementers’ lack of commitment to pursue NHIS’ equity goal.

**Conclusion:**

Inequity in healthcare access persists because of the social and institutional environment in which the NHIS operates. There is a need to effectively engage stakeholders to develop interventions to ensure that the poor are included in the NHIS.

## Résumé

**Contexte:**

Le régime national d’assurance maladie (NHIS) a été introduit au Ghana pour assurer l’équité dans l’accès aux soins de santé. Actuellement, certains pays à faible revenu et intermédiaire, dont le Ghana, utilisent des Régimes sociaux d’Assurance-Santé pour réduire les inégalités dans l’accès aux soins. Au Ghana, le NHIS a été introduit pour résoudre le problème de l’inégalité dans l’accès aux soins pendant une période marquée par des régimes de frais d'utilisation. La prime a été fortement subventionnée et des exemptions prévues pour les plus pauvres. Néanmoins, des études révèlent qu’ils sont moins couverts dans le programme. Nous avons utilisé une perspective à multi-niveaux comme outil conceptuel et méthodologique pour examiner pourquoi le NHIS ne bénéficie pas les pauvres.

**Méthodes:**

Quinze communautés des régions Est et Centrale du Ghana ont été interrogées après la mise en œuvre d’un programme d’intervention de 20 mois visant à assurer que les communautés aient une connaissance suffisante des principes et prestations d’assurance-santé et à augmenter l’inscription et le taux de rétention au NHIS. Observation et entrevues détaillées ont été employées pour recueillir des informations sur les effets de l’intervention dans sept communautés sélectionnées dans la Région centrale.

**Résultats:**

Les résultats font preuve d’une nette augmentation de l’inscription à l’assurance-santé dans la population générale, mais les pauvres sont moins couverts. Des 6.790 personnes interrogées, moins de la moitié (40.3 %) étaient actuellement couverte dans le NHIS et 22.4 % ont été préalablement assurées. Les plus pauvres étaient les moins inscrits : 17.6 % plus pauvres, 31.3 % pauvres, 46.4 % riches et 44.4 % plus riches (p = 0.000). Les taux d’inscription préalables étaient de 15.4 % plus pauvres et 23.8 % plus riches (p = 0.000). Paradoxalement, la faible inscription des pauvres a été largement attribuée à leur pauvreté. La cause structurelle sous-jacente, cependant, était le manque d’engagement des décideurs et exécutants de politique à poursuivre l’objectif du NHIS qui est celui de l’équité.

**Conclusion:**

L’inégalité dans l’accès aux soins de santé persiste en raison de l’environnement social et institutionnel dans lequel le NHIS opère. Il est nécessaire d’engager les parties prenantes pour développer des interventions et assurer que les pauvres sont inclus dans le NHIS.

## Background

Inequity in healthcare access has been of grave concern to health system analysts and researchers everywhere. They argued that since user fees denied many people access to quality healthcare [1–7], equity could only be achieved through financial risk pooling [7]. The World Health Organisation (WHO) posit that pre-payment mechanisms are critical to improving access, ensure equity and quality of care in developing countries [7]. Policy makers often assume that social health insurance schemes (SHISs) would almost automatically lead to equity. But on what evidence was this argument advanced?

Presently, some low and middle income countries including Ghana, Tanzania and Taiwan are using SHISs to reduce inequity in access to healthcare. In Ghana, the National Health Insurance Scheme (NHIS) was introduced to address the problem of inequity in healthcare access in a period that was characterised by user-fee^1^ regimes. The premium is heavily subsidised and exemption provided for the poorest. The NHIS is therefore expected to reach the poorest first but studies show that alongside its success story of improving healthcare access and helping achieve better health outcomes [8–10], the poor are less covered in the scheme [11, 12]. In-depth knowledge about why the NHIS is falling short of its equity goal has not been thoroughly investigated. This article is based on a follow-up survey carried out in 15 intervention communities in March 2011 in the Central Region (CR) and Eastern Region (ER) of Ghana. In addition, key informant interviews and observation were carried out in two out of seven communities in the CR. The conclusions that are drawn in this article are not based on only survey results, observation and discussions with key informants, but also insights acquired through close reading of academic publications, policy reports and popular press.

The novel feature of this study is the engagement of local stakeholders who are critical in NHIS’ policy implementation in discussions and the focus on events at various levels. This ensured that all possible dynamics that leads to the core poor’s low enrolment in the scheme was captured and a more credible explanation why the NHIS is not reaching them as intended is provided.

The results of this critical analysis of pathways of excluding the core poor from the NHIS in the midst of exemption will generate public interest in the ongoing debate about whether the poor are adequately included in the scheme. It will also inform the design of interventions on how to improve equity in the NHIS.

### Evolution of national health insurance in Ghana

Since Ghana attained self-rule in 1952, various health financing policies from fee-free^2^, user-fees to health insurance have been introduced with the goal of ensuring equity in access to quality healthcare. Fee-free healthcare was introduced in all public health facilities as part of President Nkrumah’s socialist development agenda to ensure that all Ghanaians have access to quality healthcare. Sustaining the policy became a challenge during the economic decline in the 1960s, the Hospital Fees Regulation, which introduced user-fees, was passed in 1963 (Legislative Instrument (LI) 1277). After President Nkrumah’s overthrow in 1966, the National Liberation Council enacted the Hospital Fee Decree 360 in 1969 to raise user-fees. The fees charged were heavily subsidised and so healthcare was described as virtually free [13]. Decline in the economy in the late 1970s and 1980s led to heavy cuts in budget allocations to the health sector; resulting in consistent shortfall in drugs, inadequate equipment and other consumables in public health facilities [14, 15].

The government of the Provisional National Defence Council (PNDC) addressed these inadequacies by replacing LI 1277 with LI 1313 in 1985 [16] and introduced pay-per-service model, ‘cash and carry’^3^, in order to recover 15 % of recurrent expenditure and full-cost of drugs. Studies reported drops in utilisation ranging from 25 % to 90 %, with the greatest decline among the poor [2, 4–6, 17]. As expected, the reaction to the revelation that user-fees denied many Ghanaians especially the poor access to quality healthcare services was so strong that an alternative health financing policy was imperative. The PNDC thus contracted local and international experts to make recommendations for the establishment of a national health insurance scheme. This culminated in the launch of a pilot NHIS by the National Democratic Congress (NDC) government in four districts in the ER (New Juabeng, Birim South, Kwawu South and Suhum Kraboah Coaltar) in 1997. These schemes stalled but ignited debates on the need to replace user-fees with health insurance. This led to the creation of community-based health insurance schemes (CBHISs) by religious groups and local government administrations. The success of many of the CBHISs [18] and the continuous pressure by local and international institutions to establish a national health insurance inspired the government to commission the Social Security and National Insurance Trust (SSNIT)^4^ to plan another centralised health insurance scheme. But this vision could not materialise due to a change in government.

The New Patriotic Party (NPP) then in opposition made a commitment in their manifesto for the 2000 election to implement a national health insurance. Upon winning the election and assuming office on January 7 2001, set up a Ministerial Health Financing Task Force to provide advice on how to develop appropriate health insurance legislation and financing mechanism. After a troubled start they came out with recommendations which led to the passage of a new National Health Insurance (NHI) Law (Act 650) under a certificate of urgency in 2003 [19, 20]. The NHIS became operational in March 2004 with the goal of replacing cash and carry, ensure equitable and universal access to a good quality healthcare within five years. The NHI Act specifies a minimum package that covers 95 % of diseases reported in health facilities in Ghana and requires no co-payment.

Since the NHIS is intended to be pro-poor, a National Health Insurance Fund (NHIF) was established to subsidise the premium of informal sector workers and pay premium for vulnerable groups including indigents referred to in this paper as ‘poorest’ or ‘core poor’, children below 18 years, SSNIT pensioners and non-SSNIT pensioners’ 70 years and above. Pregnant women were added to the exemption group in June 2008. The NHIF was financed through money allocated by Parliament, 2.5 % of the SSNIT contributions, 2.5 % value added tax on selected goods and services and the government’s annual budgetary allocation to the NHIA. Studies have reported that NHIS’ improved access to healthcare [9, 10]. Durairaj and others reported that the NHIS provides social and financial protection to insured patients and contributes to a decline in hospital deaths among the insured due to early treatment [9].

### Theoretical orientation of the study

This study, the anthropological component of a multidisciplinary research project, examines why the NHIS is not reaching the poor as envisaged. It is situated within critical medical anthropology which emphasises that researchers have academic and moral obligation to reveal and address the broad socio-economic, cultural and political contexts in which health inequities occur [21–23]. Other researchers added that health policy decisions should consider what happens at local levels in order to capture the perspectives of the targets of the policy [24, 25]. These authors proposed the use of a ‘multi-level perspective’ (MLP) in the study of health issues. The MLP emphasises that to understand a social phenomenon, information should be gathered from actors and events at various levels of social organisation. This means researchers should consider the wider context in analysing health issues and focus on different actors and associated events at various levels instead of concentrating on one level or actor. Van der Geest and others advocated the use of the MLP in the study of health policies to enable researchers capture the views of people most affected by those policies [24].

The word ‘level’ is used as a metaphor to refer to international, national, regional and local tiers of social organisation. The authors argue that events and actors’ varied interest at these levels interact to affect a social phenomenon. So researchers who confine their work to one level and or actor and ignore what happens at other levels gained limited information. They miss what happens at these levels which may be of crucial relevance to what takes place at the level they focus on. They applied the MLP to examine why Primary Health Care (PHC) did not work in many countries as intended. They showed how governments, health professionals and community members had conflicting interests in the PHC. Governments were interested in the PHC because it provides cheap healthcare services and advance their political interest. Health professionals disliked PHC because of a lack of financial opportunities. Local people on their part, perceived the PHC as second-rate healthcare. The authors thus provided a comprehensive explanation why PHC’s goal of providing health for all by the year 2000 was not achieved and many countries still have poor health indicators. Similarly, we argue that stakeholders’ behaviour and interest often conflict with the NHIS’ policy goals and make it difficult to achieve its equity goal. The MLP thus provides the appropriate conceptual framework to conceptualise the NHIS as a health financing policy that involves various actors (politicians, policy makers, implementers and community members) at different levels of the health system [24]. The use of the MLP as a conceptual framework was based on the assumption that the NHIS was introduced as a social protection initiative whose success depends on the behaviour, interest and commitment of policy makers, implementers and consumers of healthcare. Linking local events and practices to national level ideas pointing out conflicting interests that account for what happens, is the key objective of the MLP that was adopted in the project.

The MLP is also used as a methodological tool to engage local actors to explore factors that undermine the achievement of NHIS’s equity goal. This is grounded on the argument that when key stakeholders at different levels are engaged in studying a social phenomenon, in this case low enrolment of the core poor in the NHIS, its different dimensions is captured.

## Methods

### Study design and setting

This article is an analytic reflection on a large research project, about health insurance enrolment and membership renewal in Southern Ghana. The results of a baseline survey carried out in one randomly selected communities in each of the 30 districts with DHIS offices in both regions were used to form 15 intervention groups (7 in CR and 8 in ER) and 15 control (6 in CR and 9 in CR). The two regions were strategically chosen to represent the ecology (forest and coastal) and the main economic activities (farming and fishing) of Southern Ghana. They also have similar poverty incidence: 0.40 and 0.45 respectively [26].

We established a multi-stakeholder problem-solving programme (MSPSP) in the 15 studied communities. The MSPSP set up problem-solving groups (PSGs). The PSGs carried out educational and promotional activities and ensured all residents have adequate knowledge about NHIS’ operational principles and benefit package. The PSG members were selected from the health services, the DHIS and the community.

The intervention communities were exposed to 20-monthly awareness-raising campaign on the operational guidelines of the NHIS and benefits of health insurance through ‘PSG discussion groups’ at community level. The groups met every month to discuss the obstacles to enrolment and how to improve the effectiveness of health insurance with the goal of improving NHIS enrolment and retention rates (see Kotoh for detailed description of PSGs’ formation and activities) [27].

### Data collection and analysis

Quantitative data was obtained from the follow-up survey carried out in the 15 intervention communities using structured questionnaire administered to randomly selected heads of households who provided information on all their members. The data includes individual health insurance status and socio-economic status (SES). Health insurance status was categorised into three: (1) currently insured (the population that have valid NHIS cards), (2) previously insured (the population that have not renewed their membership and (3) never insured (the population that have never enrolled in the scheme).

Poverty was estimated using households’ detailed monthly consumption expenditures on food and non-food items including imputed values for items produced by the households such as food and housing to have uniformity with the 2005 Ghana Living Standard Survey (GLSS V) data. The GLSS V is an internationally accepted method for estimating household income in developing countries [25]. The households were ranked into five socio-economic categories: core poor, poor, average, rich and very rich. Calculation of the SES was done by the health economists in the project team and reported elsewhere [28, 29]. All analysis were done in SPSS version 20.

The anthropological-qualitative research was carried out in the seven intervention communities in the CR from June to September 2011 to complement and further explore the quantitative results. Two communities out of these seven received extra attention through continuous participant observation and in-depth interviews by the first author using a purposeful sample that is representative of the three local stakeholders. Forty key informants; 20 from each study site (11 community members, 7 health providers and 2 DHIS staff) were purposely selected (Table 1).

**Table 1:** Profile of key informants in each case study community

Stakeholders	Position/employment	Number
Community members	Fishermen or farmers,	3
	Traders, farmers, unemployed	3
	Traditional and opinion leaders	3
	Religious leaders	2
Health facility staff	Staff nurse	1
	Community health nurse	1
	Midwife	1
	Medical assistant	1
	Physician	1
District health Directorate staff	Disease control officer	1
	District director of health	1
District Health Insurance Scheme staff	Public Relations Officer	1
	Manager	1
Total		20

Community members were selected a few weeks into the fieldwork based on their representativeness of the target population: being 18 years and above, currently insured, previously insured and never insured as well as poor and rich individuals. Key informants who were health providers or DHIS staff were purposely selected based on their work schedule and insight into the NHIS’ operations.

The methodological features of indepth interview and observation make them extremely useful in policy research. It has the advantage of providing non-linguistic data. By entering into a direct dialogue with stakeholders the researcher observes their body language and gestures, ask further questions and probes for additional information to gain more insight into perspectives, experiences and practices to confirm the findings. For instance, observing the countenance of the DHIS staff during interviews provided additional information to support what others had said about the reasons why the care poor were less covered in the NHIS.

The qualitative data was analysed manually. First, observation and conversation notes were elaborated into field note books at the end of each day. Interviews were digitally recorded and transcribed verbatim. The transcript and observation notes were read several times to get a sense of the entire data. Second, the test was subjected to a qualitative content analysis to generate common themes emanating from the data [30]. Third, typical comments by the three categories of participants (community members, health providers and DHIS staff) were abridged into meaningful summary statements and further condensed to form themes. The themes were: local conditions of the poor, implementation of the exemption, reasons for not granting exemption and stakeholders’ interest in the NHIS. The summary statements were categorised and placed under the column created for community members, health providers and DHIS staff and put below each theme. Fourth, the second author critically reviewed the transcription and the analysis. After this, we met and discussed the entire results to ensure that the themes and summary statements reflect participants’ views. The text was referred to when a deeper understanding of an issue and the quantitative results was needed in discussing the results. Finally, we place the findings in the wider context of national politics and administrative practices, linked local events to the national level ideas and practices and pointed out stakeholders’ conflicting interests that contributed to the poor’s low coverage in the NHIS.

The Ghana Health Service Ethic Committee approved the research protocol. The consent form stated the aim of the study, participants’ right to decline to participate or opt out at any time. Participants’ confidentiality was guaranteed by not sharing their personal information with any other person.

## Results and discussion

### Descriptive statistics of households and individuals covered by the survey

The quantitative data covered 1562 households and 6790 individuals. Almost half (46.3 %) of individuals were below 18 years and 4 % aged 70 years and above. A little over half (52.5 %) were females. About 20.5 % of the population belonged to the poorest socio-economic quintiles (Table 2).

**Table 2:** Background characteristics of households and individuals covered in the survey

Overall (all household members) N = 6790	Percentages
Age	n = 6790
0-17	46.3
18-69	49.7
70+	4.0
Sex	n = 6790
Male	47.5
Female	52.5
Highest level of education of heads of households	N = 1562
None	26.3
Primary	20.6
Secondary	44.4
Tertiary	8.7
Marital status of heads of households	N = 1562
Single	15.9
Married	62.7
Divorce	21.4
Socio-economic categories	n = 6790
Poorest	20.5
Poor	20.1
Average	19.7
Rich	20.6
Richest	19.1

The findings show that the poor are less covered in the NHIS. Of the 6790 individuals covered in the follow-up survey in intervention communities, less than half (40.3 %) were currently insured and 22.4 % were previously insured and 37 % had never enrolled. There were notable differences in NHIS status across socio-economic categories with the poorest being the least covered: poorest 17.6 %, poor 31.3 %, rich 46.4 % and richest 44.4 % (p = 0.000). However, previous enrolment rate was higher among the richest: rich (23.7 %), richest (23.8 %) and poor (18.4 %), poorest (15.4 %) (p = 0.000) (Table 3). This means that more poor renew their membership regularly compared to the rich. Critical analysis of the qualitative data brought to the fore two main factors: poverty and policy makers’ and implementers’ lack of commitment to the NHIS’s equity agenda.

**Table 3:** Health insurance status by socio-economic quintiles

	N = 6790	Currently insured	*P*-value	Previously insured	P-value
Overall		40.3		22.4	
Socio-economic categories	N = 6790				
Poorest	20.5	17.6	0.000	15.4	0.000
Poor	20.1	31.3		18.4	
Average	19.7	35.0		22.1	
Rich	20.6	46.4		23.7	
Richest	19.1	44.4		23.8	

### Poverty and enrolment in Ghana’s National Health Insurance Scheme

Equity in coverage is at the heart of the NHIS’ policy. Yet our findings show that the poor are less covered in the scheme. The lower enrolment among the poor supports previous studies that enrolment in the NHIS increases with income [11, 12]. In their study in a rural district of Southern Ghana, Sarpong and others found that 38 % of households surveyed were currently enrolled in the NHIS. Of this, 21 %, 43 % and 60 % were of poor, middle and high SES households respectively [12]. Our results show that poverty affects enrolment but is less significant in membership renewal. Thus the poor when enrolled were more likely to remain in the scheme. This suggests that the poor value health insurance more than the rich. The question then is: why is the poor the least enrolled in the NHIS? Our results and evidence from earlier studies indicate that many healthcare interventions, targeted at the poor, do not often reach them as planned is overwhelming [31–37].

When community members who were considered to be core poor by local standards were asked why they had not enrolled in the NHIS or had not renewed their membership, poverty, “No money to pay premium”, was most frequently mentioned. A community leader summarised the situation of the core poor as follows: “They are very poor individuals who have no stable source of income. They struggle to get one meal a day and cannot afford the cost of premium.”

A health provider also described the situation as follows:

The good thing about the NHIS is that the insured patients are coming to the hospital early with less complicated cases. But people who are very poor come to the hospital without insurance and can’t pay their bills so we refer them to the DHIS for exemption to enable us get our money.

### Lack of commitment to achieve equity in health insurance coverage

Our results brought to the fore the underlying, less understood causes of the NHIS’s failure to achieve one of its primary objectives: protecting Ghana’s most vulnerable citizens (the core poor) against the disastrous consequences of ill health. Though policy makers’ clearly stated their commitment to bring about equity in healthcare access within five years of NHIS’ introduction through exemptions almost a decade into its operations, the core poor were the least enrolled. The exemption is not reaching them, thus contradicting policy makers’ claim that the NHIS would bring about equity in healthcare access within five years.

First, critical analysis of the criteria for exemption show that one of the conditions policy makers set for granting exemption to the core poor does not reflect local conditions of poverty. The following are the criteria stipulated in the National Health Insurance Regulations 2004, LI 1809:58 for identifying the core poor:

1. A person shall not be classified as an indigent under a district scheme unless that person
  - is unemployed and has no visible source of income;
  - does not have a fixed place of residence according to standards determined by the DHIS;
  - does not live with a person who is employed and who has a fixed place of residence;
  - does not have any identifiable consistent support from another person.
2. The conditions under sub-regulation (1) for ascertaining who is an indigent shall be incorporated in the registration form of a district scheme.
3. A person assigned the duty by a district scheme of registering persons for the scheme, shall elicit the information required under the sub regulation (1) for the classification of indigents as part of the registration process.
4. Every district scheme shall keep and publish a list of indigents in its area of operation and submit the list to the NHIA for validation [38].

Though all these criteria are restrictive, the 1b criterion which DHISs referred to as homelessness, defined as lacking a roof and not having any one to provide care, to qualify the core poor for premium exemption [39], disqualifies almost everybody. The reality is that homelessness is nearly non-existent in the districts. The core poor are described as persons afflicted with ‘*ohia buburoo’* (abject poverty) who do not have a stable source of income to enrol their household members and renew their membership regularly. In farming communities, a number of them were engaged as labourers by farmers to sow and harvest crops. They were also engaged by boat owners during the bumper harvest in fishing communities. Some did menial jobs and did not have a stable income while others were totally unemployed and usually lived on the occasional benevolence of family members or friends. The core poor normally live in family houses, with friends or in dilapidated houses. Apart from the cities, homelessness does not exist. I did not find a normal homeless person in the 15 communities I visited during my fieldwork. Everybody including the core poor had a home. Homelessness is mostly a characteristic of mentally disturbed people who roam cities and towns. In her study of community concepts of poverty in the CR of Ghana, Aryeetey and her colleagues also observed that homelessness is an inappropriate condition for granting exemption to the poor [28, 40]. As has been observed, the effectiveness of SHISs is the ability to reduce genuine exclusion [41]. We therefore question the motive for setting a criterion which disqualifies almost all potential beneficiaries as the basis for granting the core poor exemption.

The relevant questions are: If the poor are the primary target of the NHIS, why set a criterion that excludes them? Whose definition of the core poor should count: the one by policy makers or the one by the community? Analysis of these questions reveals insights regarding the motivation to establish a criterion that eliminates potential beneficiaries: lack of commitment. If policy makers were genuinely committed to exempting the core poor, they would have ensured that all the criteria reflect the conditions of the target group and guide collectors on how to register them. Why these actions were not taken is explained by looking at the political situation at the time that the NHIS was introduced and taking into account the financial implications of granting exemptions to all those who would qualify. Enrolling all the exempt categories would have depleted the country’s budget so they needed a definition that would drastically reduce the financial burden without changing the election slogans.

At the time the policy was introduced and even now the NHIS has a large exemption group (more than half of Ghana’s population). The 2003 Ghana Demographic Health Survey reported that 44 % of Ghana’s population was below 15 years [42]. The 2000 Population Census showed that 5.3 % were above 64 years [43]. The 2010 Population and Housing Census also showed that 30.3 % and 4.7 % of the population were under 15 years and 65 years and above respectively [44]. These nation-wide figures roughly agree with our 2011 survey results which show that children (0-17 years) form 48.1 % of the population and 3.4 % was above 70 years. In the case of the core poor, the Ghana Living Standard Survey shows that about a third (28.5 %) of Ghana’s population lived below the poverty line [26]. Considering the core poor alone, it means that the government would have to pay premiums for about 2.5 million people. This equals a total cost of about GH₵35 million (US$25 million) per annum, which would be a significant demand on the country’s budget. For a country already overstretched with unfulfilled needs in other sectors, such as education and roads, the money to cover all these exemptions was simply not available. The NHIA debt to health facilities is an indication of the huge financial burden of exemptions on the country’s limited financial resources. By the end of 2008, the NHIA owed health facilities about US$34 million [36].

The NHIS was introduced nation-wide in March 2004 and elections were held in December of the same year. The homelessness criterion thus seemed a strategy to lessen the financial burden of enrolling all core poor while serving as propaganda to accumulate political capital for the pending election. There was nothing to lose if in practice the homelessness criterion eliminated almost all potential beneficiaries while appearing to be fulfilling the government’s moral obligation to the poor and also showed the international community they were committed to ensuring the poor have access to healthcare. Also, the NHIS policy-making process was characterised by political rhetoric [19, 45]. What was important at that time was to win votes and not practicalities of implementing the exemption policy. If this had not been the case, and politicians were truly committed they could have ensured that all the criteria set reflect the reality of the core poor. Poverty needs to be defined by the community. This view starts from the assumption that opinion and community leaders understand local conditions of poverty and are in a better position to devise effective guidelines that could be used to identify them. A critical analysis of various methods of identifying poor households, concluded that the community criteria of classifying the poorest members correlated with mean testing and the proxy mean testing considered as the gold standard [28]. One can hardly reach another conclusion than that the realities of implementing the exemption policy for the poor was intentionally disregarded. As pointed out: “A policy is only as good as its implementation arrangement.” [46].

First, policy makers recognised that no matter how low the cost of premium, the core poor cannot pay so provided exemption to ensure their inclusion in the NHIS. But the exemption is not reaching them.

When DHIS staff and collectors were asked why they did not enrol the core poor, they demonstrated lack of commitment to the equity goal when discussing the issue.. Their first response was normally the problem of identification. A DHIS staff explained why they hardly exempt the core poor from paying premium as follows: “*The main criterion we use is homelessness which disqualifies almost everybody. But we occasionally give exemptions when health providers refer patients who cannot pay their hospital bill to us*.” Critical observation of their countenance and further discussions with them revealed that it is not the problem of identification but their answer was often a convenient and morally acceptable excuse. Thus just like previous exemption policies which were not successful, only a few core poor benefit from exemptions under the NHIS. The core poor are unable to claim the exemption they are entitled to. How is this possible?

Contrary to the general opinion that inadequate exemption for the core poor is mainly due to identification difficulties, we argue that the explanation runs deeper. The DHIISs could have used local indicators of poverty such as unemployment, no visible source of income and consistent support from another person which share commonalities with what is stated in the NHIS policy (1a and 1d). Ignoring these community indicators of poverty and using homelessness as the decisive criterion proved a convenient tool to exclude nearly all core poor without seeming unreasonable.

Also, it was observed that DHISs’ often undertake revenue generation activities but virtually do nothing to identify the core poor for exemption. Throughout my fieldwork I did not see the DHISs organising any activity to identify the core poor for premium exemptions. The following comment by a DHIS staff illustrates their attitude towards exemptions for the core poor: *We need money so if we go to communities and tell them about exemption for the core poor, how do we get revenue to meet some of our expenditure?*

Additionally, collectors who were expected to recommend the core poor to the DHISs to be certified as qualified for exemption usually do not disseminate information about exemptions. They focus mainly on premium collection. One of them said: “*I am not paid for enrolling those who do not pay premium. So if I continue registering them how do I get money for the work I’m doing.”* These comments and similar others show clearly that collectors’ attitude may have to do with the fact that they were not paid for registering the ‘exempt group’. This practice defeats the purpose of the NHIS as a safety net, which is expected to provide the poor with access to healthcare when ill and not when they are unable to pay for their healthcare. One can thus conclude that DHISs’ staff’s decision not to pursue NHIS’ equity agenda was often based on the financial implications of exemptions for their offices. The premium contributes significantly to their internally generated fund (IGF) which they need to meet some of their recurrent expenditure. The IGF is also used to judge the performance of DHISs. These created disincentives to exempt people especially the core poor whose endorsement solely depends on the staff.

Further, many community members told me they were not even aware of the exemption policy. This supports an earlier study on exemption across 18 communities in the Northern, Upper East and Upper West Regions of Ghana also found that 61 % of their respondents did not know about exemptions for the poor [37]. A study found that exemptions for indigents (core poor) under Ghana’s NHIS were 1 % in 2008 [36]. In their study on exemptions of Community Health Fund in Tanzania observed that the managers often refuse request for exemptions because they felt it reduces their revenue [34].

These observations clearly indicate lack of interest from the DHIS staff and collectors to pursue NHIS’s equity agenda. Thus NHIS’ purpose as a safety net, which is expected to ensure equity in healthcare access, is not achieving its objective. Thus macro-level policies targeted at the poor often fail to achieve their objectives because of implementers’ attitudes.

We draw on Lipsky’s concept of street-level bureaucrats’ to explain the gap between the exemption policy and implementation. In his analysis of frontline public service workers’ behaviour in the United States of America [47]. He observes that they generally display a high margin of discretion and their actions effectively become public policy rather than the objectives of the documents developed at the policy level. Similarly, in this study DHIS staff and collectors can be described as stress-level bureaucrats who used their discretion and decide which aspects of the NHIS policy needs to be pursued: revenue generation or exemption. As has been pointed out by earlier researchers, there should be trade-offs to achieve both goals [48]. We found that DHISs’ staff and collectors, who are to pursue both goals, have gradually shifted their focus in favour of revenue generation. They were more concerned about increasing their IGF to enhance their image and not to vigorously look for people to exempt since giving exemptions means they lose revenue.

The argument that policy makers and implementers are not committed to pursuing the NHIS’ equity agenda is strengthened when one considers the fact that the non-applicability of the homelessness criterion has been discussed in the public domain since the NHIS was introduced, yet no solution is in sight. This is not to say that nothing is being done to improve the applicability of the exemption policy, but the moral urgency required of both past and present governments seems to be lacking.

The amended NHI Law, Act 852 section 29 (d) states that ‘a person classified by the Minister responsible for Social Welfare as an indigent (core poor), qualifies for exemption and are enrolled in the NHIS [49]. With this, the NHIA depends on the Livelihood Empowerment against Poverty (LEAP)^5^ Programme to identify the core poor. The LEAP started in March 2008 by the Government of Ghana as a social cash transfer programme to vulnerable households across the country. It is still in its trial phase and has reached only 35,000 individuals [50]. Eligibility is based on poverty and having a household member in at least one of these three demographic categories: single parent with orphan or vulnerable child, elderly poor, or person with extreme disability and unable to work. Initial selection of households is done through a community-based process and verified centrally with a proxy means test. It must be noted that none of the core poor covered by the project in intervention communities in March 2011, benefited from the LEAP programme. This suggest that despite the improvement in the exemption criteria, it still does not adequately resolve the problem of excluding the poor from the scheme. Thus the LEAP though laudable in its current form seem not to be the panacea to the exclusion of majority of the poor in the NHIS.

These reflections clearly show that even if the implementation arena is littered with other barriers, policy makers’ professed goal of ensuring equity in healthcare access is not being given the urgency it requires to ensure all core poor are included in the NHIS.

### Limitation of the study

Since the study was conducted in only two regions in Southern Ghana, the NHIS’ implementation contest and dynamics might not be exactly the same in the rest of the country. Notwithstanding this limitation, we believe the inclusion of events at the national level make our findings reflect the enrolment of the poor in the NHIS and the reasons for their low coverage in the whole of Ghana. That said, the conclusions should be interpreted with care.

## Conclusion

This study has shown that though the NHIS is a social security programme targeted at the poor, the socio-economic and political context in which it operates exclude them from the scheme. After almost ten years into its operations, mere 18 % core poor in the sampled population have a valid NHIS card even though they value health insurance. We recommend that community leaders should be engaged to develop criteria that reflect local conditions of poverty. They understand local conditions of poverty and are in a better position to devise effective guidelines for identifying the poorest members instead of policy makers who might not be familiar with local situations.

Our results show that financial implications of exemptions to DHISs and collectors contribute to exclusion of the core poor from the NHIS. Since the economic cost of prolonged illness does not affect the individual but also the country’s development, we therefore suggest that politicians and policy makers should be conscientised to appreciate that it is economically prudent to provide DHISs adequate funds to reduce their reliance on the IGF and pay collectors for enroling the core poor to motivate them to pursue the NHIS’s equity agenda.

Moreover, the majority of the core poor are excluded from the NHIS essentially because they could not demand exemption provided them. They are the silent ones in society who cannot challenge policy makers and DHISs for not granting them exemptions for which they qualify. We thus suggest that the powerful in society such as researchers and civil society organisations should advocate for them, point out the gaps in the exemption policy and project how the interest of politicians, DHISs’ and collectors undermines the attainment of NHIS’ equity goal. This will put pressure on the government to develop appropriate exemption implementation strategies to ensure the poorest of Ghana’s population, the main target of the NHIS, are covered.

## Footnotes

1 User-fees refer to out-of-pocket payments for some healthcare services at the point of utilisation.

2 Fee-free refers to free access to healthcare for all residents in Ghana at public facilities.

3 Cash and carry was a WHO and UNICEF initiative adopted by African Health Ministers in Bamako, Mali, in 1987. The policy aimed at improving drug supplies in public health facilities and led to out-of-pocket payment for full cost of drugs in public health facilities.

4 The Social Security and National Insurance Trust (SSNIT) is a government pension scheme in Ghana that most formal sector workers and their employers contribute to.

5 The Livelihood Empowerment against Poverty (LEAP) Programme started in March 2008 and expanded in 2009 and 2010 as a social cash transfer programme which provides cash and health insurance to extremely poor households across Ghana. It is funded by the Government of Ghana (50 %), donations from the Department for International Development and a loan from the World Bank and implemented by the Department of Social Welfare in the Ministry of Gender, Children and Social Protection.
